# Strategies to prevent postoperative delirium: a comprehensive evaluation of anesthesia selection and drug intervention

**DOI:** 10.3389/fpsyt.2024.1518460

**Published:** 2024-12-23

**Authors:** Shaoze Lan, Shuai Liang, Huiwen Wu, Shihao Deng, Kenan Sun, Canming Ye, Liu Yang, Lunzhu Ciren, Jun Li

**Affiliations:** ^1^ Department of Orthopedics, The Second Hospital of Anhui Medical University, Hefei, China; ^2^ Department of Orthopedics, Shannan City People’s Hospital, Shannan, China; ^3^ Institute of Orthopedics, Research Center for Translational Medicine, The Second Hospital of Anhui Medical University, Hefei, China

**Keywords:** delirium, perioperative medication, anesthesia, hip fractures, prevention, treatment

## Abstract

Postoperative delirium (POD) represents a common neurological complication encountered predominantly among the elderly cohort undergoing surgical intervention for hip fractures. This phenomenon, particularly commonplace in geriatric populations with heightened preoperative risk profiles, pronounced comorbidities, and later stages of lifespan, poses complex clinical challenges. The impact of perioperative pharmacological interventions and anesthetic strategies on POD’s emergence cannot be understated, as it may profoundly affect the length of hospital stays, rehabilitation milestones, and the overall mortality hazard. The pharmacotherapeutic landscape for managing POD remains constrained, underscoring the imperative nature of preventive measures. Prudent preoperative risk stratification, meticulous intraoperative neuromonitoring guided by electroencephalographic studies, and a holistic postoperative patient welfare model are cornerstone recommendations in the quest to mitigate POD’s incidence. Nonetheless, an extensive exploration into the influence of anesthetic approaches and perioperative medications on the emergence of POD is yet to be satisfactorily charted. Our investigation endeavors to dissect the nexus between anesthetic modalities, perioperative pharmacological interventions, and POD incident rates among the elderly with hip fractures. This study spotlights pivotal determinants of POD in the wake of hip fracture surgery by evaluating and synthesizing data from peer-reviewed sources that adhere to rigorous inclusion criteria. Preliminary studies have revealed that certain anesthesia protocols and perioperative medications may increase the potential incidence of POD, such as higher depth of anesthesia or benzodiazepine use, and the incidence of POD in specific populations, such as patients with higher age, prior history of psychosis, and lower intraoperative oxygen saturation The findings from this study are instrumental in refining strategic perioperative plans tailored for the elderly recipients of hip fracture surgery, aimed at not only diminishing the incidence but also the gravity of POD. Despite these forward steps, the clinical uncertainty concerning the efficacy and safety of the specific drugs and surgical techniques in question remains. These lingering questions underscore the exigency for more extensive, empirically grounded research to consolidate the learnings of this investigation.

## Highlights

- Most of the studies reviewed in this paper found no statistical difference in the effects of local and general anesthesia on postoperative delirium.
- There is a significant correlation between the use of benzodiazepines and postoperative delirium.
- The level of sedation affects the occurrence of postoperative delirium.
- Melatonin seems to be effective in preventing postoperative delirium.
- Based on the comparison of a large number of kinds of literature, it is concluded that the influence of perioperative drugs and anesthesia on postoperative delirium is not uniform.

## 1 Introduction

**Figure 1:**
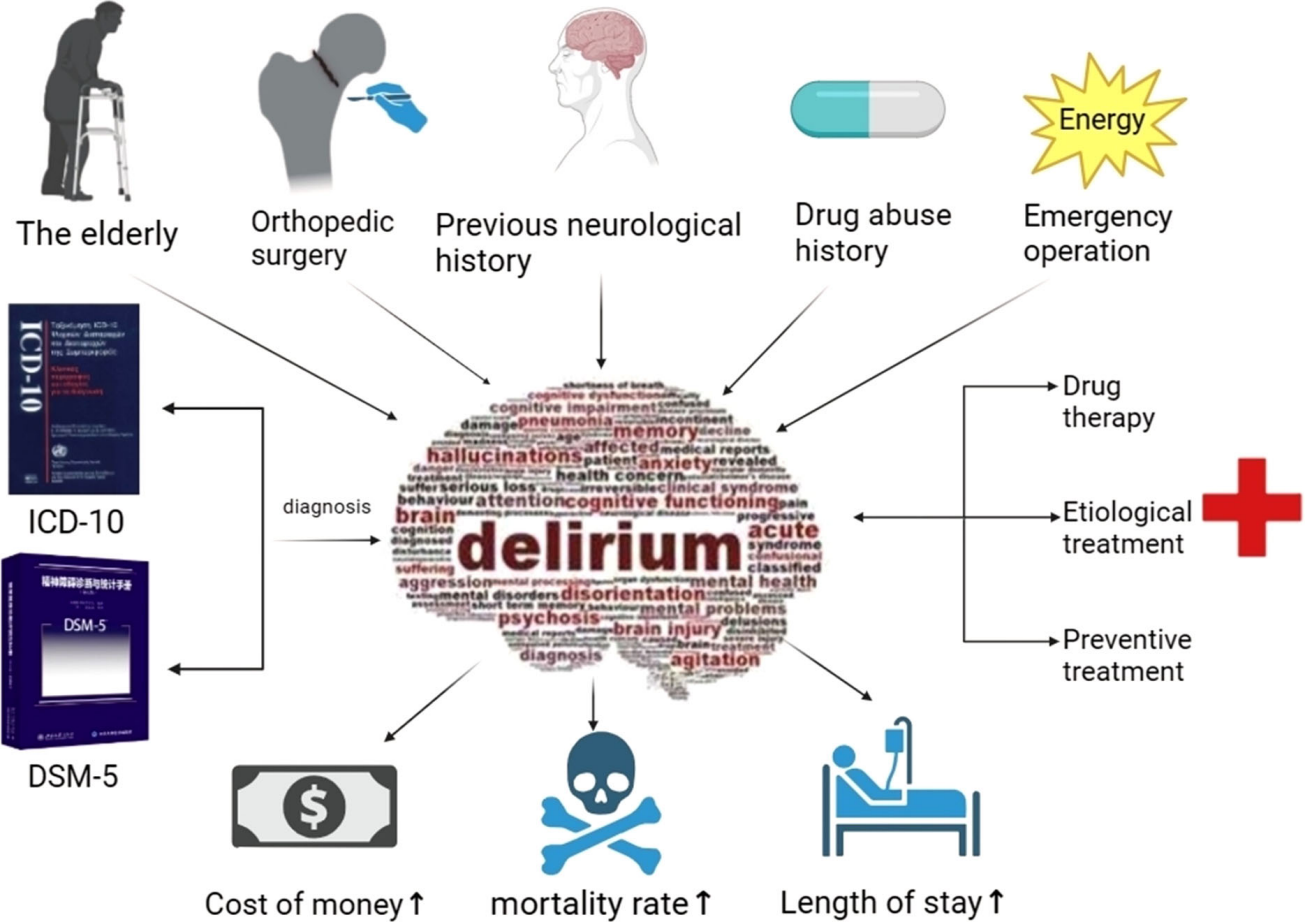
Provoking factors in delirium pathogenesis, the ultimate diagnostic benchmark, prognostic indices, and management strategies.

Postoperative delirium (POD) is a common clinical occurrence following hip fracture surgery, particularly among the elderly patient population (1, 2). Around one-quarter of these patients experience POD, which is a syndrome that often arises alongside other medical conditions and physical declines that are inherent to an aging demographic (3, 4). Delirium not only causes significant cognitive disturbances in attention, cognition, and orientation but also leads to prolonged hospital stays and increased short-term and long-term mortality rates for patients. This places additional physical, mental, and economic burdens on both patients and their families (5–7) **Figure 1**. Despite the fact that delirium after hip fracture is so common (8), the exact pathophysiological mechanisms remain to be conclusively elucidated (9), prevailing assumptions among researchers propose that the onset of delirium could be associated with factors such as neuroinflammation, dysfunction of the neuroendocrine system, augmented level of reactive oxidative stress (ROS), and aberrations in neurotransmitter functioning (10). A comprehensive understanding of its pathophysiology is crucial for the development of extensive preventative strategies and treatment approaches.

Non-pharmacological interventions have recently gained considerable attention in addressing POD (11). However, it is important not to dismiss the role of traditional pharmacological interventions and the significance of anesthetic drugs and perioperative drug support in the occurrence of delirium (12). In a study examining a population over 80 years of age hospitalized for hip fractures, multivariate logistic regression analysis demonstrated a correlation between the types and doses of drugs administered with the incidence of delirium (12). Given the instrumental role of anesthetic and perioperative medications in hip surgery, there is promise in mitigating this prevalent health issue through stringent oversight in medicinal administration. This emphasizes the importance of continuous evaluation of surgical procedures, anesthetic techniques, and pharmaceutical treatments in decreasing the prevalence of POD following hip fracture surgery.

This article reviews the effects of perioperative drug therapy, anesthesia methods, and anesthetic drugs on POD in elderly patients with hip fractures, to provide effective and feasible strategies for the prevention and treatment of POD in elderly patients with hip fractures.

## 2 Mechanism of delirium pathology

**Figure 2:**
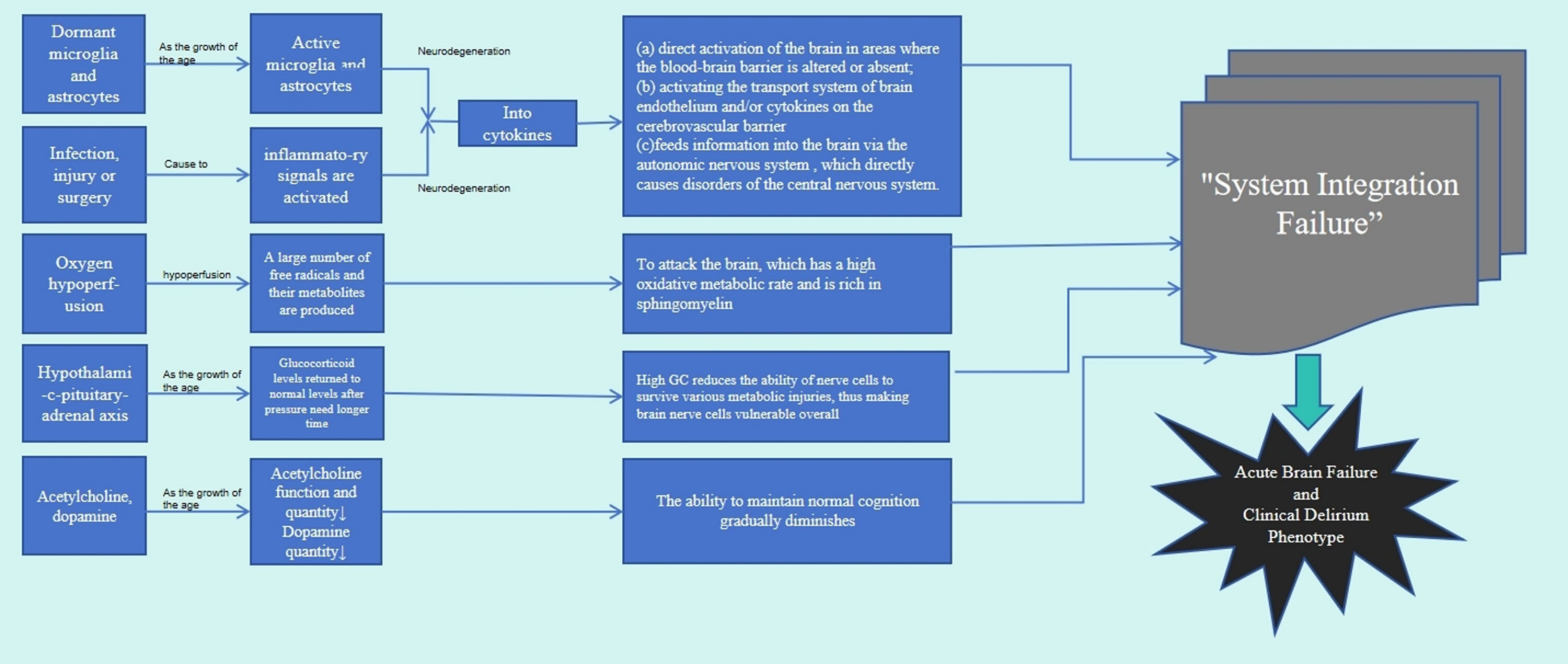
Four ways are down the nerve inflammation theory, theory of neuroendocrine dysfunction, reactive oxidative stress theory, and theory of neurotransmitter disorders. Through the interaction between the four approaches, the theory of System Integration Failure is finally obtained.

Currently, the intricate pathogenesis underlying POD following hip fracture in geriatric patients remains to be fully comprehended (9). Current research focuses on neuroinflammation, dysfunction of the neuroendocrine system, elevated levels of reactive oxidative stress (ROS), and abnormal neurotransmitter function (10). It appears improbable for a single proposed theory to comprehensively explicate either the etiological or the phenomenological elucidations of delirium. Instead, it is more plausible that the collaborative function of two or more theories accounts for the intricate cognitive and behavioral alterations that result in the ultimate biochemical aberrations, subsequently leading to delirium. To create a comprehensive understanding of intertwining these crucial theories, a novel theory termed “System Integration Failure” has been proposed (**Figure 2**). It delineates the varying contributions of each theory to present an intricate network, underscoring the intersections and reciprocalities. Additionally, it explicates how discrepancies in these contributions could contribute to the progression of delirium (13). Further exploration of the pathophysiological mechanism of delirium is expected to provide the scientific basis for formulating more effective prevention and management strategies. Future research will help uncover the deep causes of delirium development, thereby driving improvement and innovation in clinical practice.

## 3 Epidemiology and Outcomes of POD

The incidence of postoperative hip fracture delirium exhibits considerable variability owing to a diversity of factors including patient demographics, surgical interventions, diagnostic methodologies, timeframes of assessment, and research designs. In the general adult population, the prevalence of POD falls within a range of 2.6% to 4.3% (14, 15). This incidence escalates markedly in older cohorts; individuals aged 60 and above experience a surge in POD rates to between 12.0% and 23.8% (16, 17). The prevalence among those over the age of 70 reaches 10.5% (15). Notably, the likelihood of delirium after hip fracture surgery amplifies drastically, with a noted increase in risk of 70-80%. Equally significant, delirium occurrence post-cardiovascular surgery is reported at 23.4% (18), while hip fracture surgery-related delirium stands at 16.9% (19). The incidence associated with emergency surgical procedures ranges from 22.7% to 26% (20, 21). Furthermore, the prevalence of delirium in patients admitted to the intensive care unit (ICU) postoperatively is documented at 24.4% (22). These data underscore the complexity of POD epidemiology and the need for multidimensional prevention and management strategies, giving contemporary clinicians new insight into the need to pay more attention to the occurrence of postoperative delirium, especially in older (> 70 years) patients with hip fractures, as well as in patients undergoing emergency surgery and IUC.

Delirium serves as an impactful prognostic indicator of adverse outcomes. In the immediate aftermath of surgery, delirium in patients has been correlated with extended hospitalizations, longer intervals to discharge, and an inflated rate of readmissions within 30 days, surpassing other major complications in its contributory role (23, 24). An emerging body of research suggests marked associations between the occurrence of delirium, amplified perioperative risk, and long-term mortality rates (3). Furthermore, delirium has been linked to a diminished health status and quality of life (25), and an elevated Alzheimer’s disease risk (23, 26). This underpins the salience of inhibitive measures against delirium, continual cognitive monitoring post-delirium, and ongoing surveillance of mortality rates in the wake of a delirium episode. Additional investigations within specific medical cohorts and Intensive Care Units are warranted. Future lines of inquiry ought to evaluate the relationship between the longevity, severity, and distinct types of delirium, and the impact on variables such as postoperative cognitive function, mortality, and dementia risk.

## 4 Risk factors for POD

Research has demonstrated that the incidence of POD in hip joint patients is associated with a multitude of factors, which can be classified into two categories: predisposing and precipitating risk factors (27) as delineated in **Table 1**. The predisposing factors encompass advanced age (28), gender (29), packed red blood cells implementation (30), occurrence of hearing loss (31), frailty (32, 33), antecedent history of mental disorders (34), renal failure (35), preoperative cognitive disability (29), lack of education (36, 37), level of serum albumin (35, 38), Charlson Comorbidity Index (CCI) (29), and high-risk assessment score (39). Precipitating factors comprise the depth of anesthesia level as monitored by brain function (40), instability of blood pressure levels (37), instance of alcohol withdrawal (39), degree of pain felt by the patient (41), medication administered perioperatively (42), the duration of mechanical ventilation (30, 34), cerebral oxygen saturation levels (43–45), the necessity for perioperative blood transfusion (46), elongated operation time (47), execution of emergency operation (48, 49), disturbances in fluid/electrolyte (34), atrial fibrillation (37), and duration of emergency room stay (34).

**Table 1:** Predisposing factors and Precipitating factors of POD and their odds ratio and 95% confidence interval (CI).

	Variables	Odds ratio (OR)	95% CI
Predisposing factors	Age	6.13	5.75, 6.54
	Gender	1.21	1.15, 1.28
	Packed red blood cells	3.00	0.90, 9.94
	Hearing loss	2.57	1.03, 6.41
	Frailty	2.14	1.43, 3.19
	Previous history of mental illness	6.84	3.21, 4.33
	Renal failure	1.4	1.24, 1.58
	Preoperative cognitive impairment	6.84	3.27, 14.33
	Undereducation	0.81	0.71, 0.92
	Serum albumin	2.45	2.01, 2.94
	CCI index	1.55	1.25, 1.92
	High-risk assessment score	2.4	1.01, 5.60
Precipitating factors	Depth of anesthesia	0.56	0.40, 0.77
	Blood pressure instability	2.73	1.16, 6.40
	Alcohol withdrawal	1.85	1.51, 2.27
	Pain	2.2	1.2, 4.0
	Perioperative medication	2.47	2.04, 2.97
	Long-term mechanical ventilation	1.1	1.04, 1.21
	Cerebral oxygen saturation	5.82	2.12, 19.2
	Perioperative blood transfusion	2.906	0.912, 9.259
	Prolonged operation time	1.06	1.03, 1.08
	Emergency operation	3.56	1.77, 7.15
	Fluid/electrolyte disturbance	4.52	2.94, 6.95
	Atrial fibrillation	2.49	1.20, 5.20
	Emergency room stay time	2.23	1.13, 4.41

A plethora of literature accentuates the correlation between the onset of POD and advanced age (18, 28, 30), thereby corroborating that the age factor stands as a pivotal element in predicting the delirium risk index. This predilection might be ascribed to the prevalence of cerebrovascular diseases as well as heightened vascular fragility in older adults (50), sluggish blood flow, and neurotransmitter irregularities (51). Maldonado et al. have indicated that post the age of 50, the probability of succumbing to delirium elevates each successive year (52). Geriatric populations constitute the majority of maintenance drug users and are the most susceptible to adverse drug reactions, which can potentially account for the pronounced incidence of delirium in this demographic (53). The classification of the patient’s status by the American Society of Anesthesiologists and other co-existing diseases also influence the occurrence of POD. Furthermore, other co-morbidities can be appraised utilizing the improved Chalson Common Disease Index (CCI) (54). A pre-existing cognitive impairment and depressive dementia also determine the development of POD in most instances (55), which might be attributed to occurring alterations in the underlying neurotransmitter, and the predilection of hypoxia and anticholinergic drugs in these patients (43, 56), despite some diverging viewpoints concerning the influence of gender on the onset of POD (18, 57), which could be a result of sampling inaccuracies. Intraoperative pain which prompts a substantial stress response or necessitates the use of analgesic drugs (29, 41), aggravates the incidence of POD. Prolonged Intensive Care Unit (ICU) stays and hearing loss escalate patient anxiety (18, 31), thereby elevating the risk of POD in the backdrop of sub-optimal postoperative care.

This paper, in light of the preceding factors, endeavors to evaluate the association between the type of anesthesia and the routinely administered perioperative drugs, while leaving the discussion of other risk factors out of its ambit.

## 5 Diagnosis of POD

The severity of postoperative delirium is not in doubt, but its prevalence is often underestimated, leading to underdiagnosis. A meta-analysis published in 2021 noted that the DSM(Diagnostic and Statistical Manual of Mental Disorders)-5 diagnostic criteria for mental disorders are particularly sensitive to screening inpatients and patients with acute illness. However, this routine screening is time-consuming and requires significant training time (58). In addition, confusion assessment (CAM) is the most widely used screening tool with high sensitivity and specificity due to various modified and adapted versions, such as short CAM and Intensive Care Unit Confusion Assessment Method (CAM-ICU); However, specific training is needed to accurately implement this (59). In contrast, the Nurse Delirium Checklist (NUDESC), developed by Gaudreau et al. (60), is a quick and easy-to-complete tool for nurses, is easy to adapt, and has proven effective in multiple studies (15), diagnostic criteria are **Table 2**. However, this article has certain limitations, similar to other meta-analysis, it may be susceptible to publication bias, and the number of studies included in this review is limited due to clinical setting criteria. Therefore, for early detection and appropriate treatment to reduce the duration, severity, and negative consequences of postoperative delirium, clinicians may also subjectively detect delirium without the use of effective tools (61). Our literature review has clarified the five typical clinical presentation areas associated with delirium, which are (a) cognitive impairments, which manifest as distorted perception, compromised memory, deficient capacity for abstract thinking, and disorientation (62); (b) attentional deficits, evidenced by a diminished consciousness and a reduced ability to focus, sustain, or shift attention (63); (c) disturbances in circadian rhythms, indicative of erratic sleep-wake cycles (64); (d) affective instability, characterized by states of confusion, anxiety, irritability, and in some cases, explicit anger (65); (e) perturbations in the stream of mental activity which can resemble Schizophrenia or Affective Disorders (66). Taking these factors into account, we can provide better treatment for POD patients.

**Table 2:** Nursing Delirium Screening Scale (NUDESC) diagnostic criteria.

Number	Content
A	Altered state of consciousness: may change from wakefulness to lethargy or lethargy, lack of concentration, slow response to questioning, blurred consciousness, and decreased ability to perceive and respond to external stimuli.
B	Behavioral changes: Symptoms of restlessness (such as frequent rise without inducement, attempts to remove catheters, break free of restraints, etc.) or excessive quietness (activity becomes less active and less responsive to external interactions).
C	Speech changes: Becomes disjointed, incoherent, speaks gibberish that is not relevant to the current situation and is difficult to steer back to normal conversation topics.
D	Orientation disorder: the existence of time (do not know day and night, time), place (do not know where the department or hospital), people (mistaken medical staff or family members) orientation disorder.
E	Hallucinations or delusions: visual hallucinations (seeing objects or people that are not present), auditory hallucinations (hearing sounds that are not present), and delusions (such as misidentifying equipment in a ward) may occur.

## 6 Anesthesia and POD

The impact of different anesthesia strategies and perioperative medications on the occurrence of POD in patients with hip fractures has been extensively studied (67–69). However, the existing research has not provided a clear evidence base from which definitive guidelines applicable to standard clinical practice can be derived (42). This manuscript will present a comprehensive data analysis, examining the influence of various anesthesia modalities and medications on POD. Due to the limited research on the incidence of POD after orthopedic surgery and anesthesia and perioperative drugs, the literature retrieved in this paper will also involve the incidence of POD after other types of surgery, so that more convincing research conclusions can be drawn.

### 6.1 Inhalational anesthesia vs. intravenous anesthesia

Through extensive literature research, scholars have presented divergent findings regarding the incidence of POD between inhalational anesthesia and intravenous anesthesia. There are two major viewpoints in this regard. Firstly, some researchers believe that there is a statistically significant difference between the incidence of POD under inhalation anesthesia and that under intravenous anesthesia (70–73). A single-center observational study involving 30,075 patients undergoing general anesthesia reported an increased risk of POD with volatile anesthetics. Another study conducted on elderly patients undergoing major cancer surgery compared propofol and sevoflurane anesthesia, and found that the incidence of POD was lower by one-third in the propofol anesthesia group (relative risk 0.68 [95% confidence interval: 0.48-0.95]; P=0.023; adjusted relative risk 0.59 [95% CI: 0.39-0.90]; P=0.014) (74). A retrospective study comparing patients aged 65 and above who received intravenous anesthesia or inhalational anesthesia in a Japanese hospital database indicated that total intravenous anesthesia (TIVA) was associated with a slightly lower incidence of POD compared to inhalational anesthesia (IA) (75). Furthermore, a meta-analysis of 12 high-quality randomized controlled trials involving 1,440 patients showed consistent results, with inhalational maintenance anesthesia associated with a higher incidence of POD compared to propofol-based intravenous maintenance anesthesia (risk ratio [RR]: 2.02; 95% confidence interval [CI]: 1.30-3.14; P = 0.002) (76).

On the contrary, the second viewpoint suggests that there is no statistically significant difference in the incidence of POD between inhalational anesthesia and intravenous anesthesia. A multicenter randomized controlled trial comparing sevoflurane with total intravenous anesthesia in patients undergoing valve replacement surgery showed no difference in the occurrence of POD between the two groups (27.8% [35/144] vs. 25.9% [35/145], 1.10, 95% CI: 0.64-1.90, p = 0.736) (77). Another randomized controlled study comparing isoflurane inhalation anesthesia (ISO) with propofol-based total intravenous anesthesia (TIVA) demonstrated no significant difference in cognition when ISO and TIVA were used for the maintenance of general anesthesia. The incidence of POD was also similar between the two anesthesia types (78). investigated the POD incidence in 100 elderly obese patients undergoing total knee replacement and reached a consistent conclusion that there was no difference in POD incidence between the desflurane and propofol groups (79).

The existing research literature supports two different views, although they are somewhat contradictory. These insights provide a strong evidence base for future prevention of POD through the selection of appropriate anesthesia methods. Further research should synthesize these different perspectives to reveal the exact association between anesthesia and POD and provide more precise guidance for clinical practice (**Table 3**).

**Table 3:** Comparison of the effect of inhalation anesthesia and intravenous anesthesia on POD.

Study	Year	Design	No. in cohort	Study protocol	Comment
Koji Ishii et al. (70)	2016	RCT (Randomized Controlled Trial)	59 participants	sevoflurane anesthesia group (n=30) VS propofol anesthesia group (n=29)	The incidence of POD in the propofol anesthesia (6.9%) was significantly less than that observed in the sevoflurane anesthesia (26.7%; 038).
Thomas Saller et al. (71)	2022	Single Center Observational Study.	30,075 participants	Delirium was assessed with the Nursing Delirium Screening Scale at the end of the recovery period.	Out of 30,075 records, 956 patients (3.2%) developed delirium in the post-anesthesia, and volatile anesthetics increased the risk for delirium in the elderly 1.8-fold compared to total intravenous anesthesia.
Shuang-Jie Cao et al. (74)	2023	A multicentre randomized trial	1194 participants	sevoflurane anesthesia group (n=597) VS propofol anesthesia group (n=597)	Delirium occurred in 8.4% (50/597) of subjects given propofol-based anesthesia vs 12.4% (74/597) of subjects given sevoflurane-based anesthesia (relative risk 0.68 [95% confidence interval (29): 0.48-0.95]; P=0.023; adjusted relative risk 0.59 [95% CI: 0.39-0.90]; P=0.014).
Miller David et al. (72)	2018	Review	28 RCTs with 4507 randomized participants	–	What little evidence there is for maintenance with propofol-based TIVA or with inhalational agents affecting incidences of postoperative delirium
Yong Yang et al. (76)	2023	A Meta-analysis	12 high-quality randomized controlled trials including 1440 patients	–	Compared with propofol-based intravenous maintenance of anesthesia, inhalational maintenance increased the incidence of ED in adults (risk ratio [RR], 2.02; 95% confidence interval [CI]: 1.30-3.14; P =0.002).
Xinchun Mei et al. (73)	2020	A randomized clinical trial study	209 participants	propofol anesthesia group (n = 106) or sevoflurane anesthesia group (n = 103)	The incidence of postoperative delirium was 33.0% with propofol anesthesia and 23.3% with sevoflurane anesthesia (p = 0.119),
Guang-You Duan et al. (77)	2022	RCT	289 participants	sevoflurane anesthesia (n = 144) or propofol-based total intravenous anesthesia (n = 145)	There was no difference in the incidence of delirium between patients receiving sevoflurane and total intravenous anesthesia (27.8% [35/144] vs. 25.9% [35/145], 1.10, 95% CI: 0.64 to 1.90, p = 0.736).
Thomas J. Farrer et al. (78)	2023	A prospective, randomized study	199 participants	inhalation anesthesia (ISO) with isoflurane(n=99) VS total intravenous anesthesia (TIVA) with propofol (n=100)	when examining ISO versus TIVA for maintenance of general anesthesia, there is no significant difference in cognition between anesthetic types
Pedro Tanaka (79)	2017	A randomized, controlled, double-blinded clinical trial	90 participants	Propofol group(n=45) VS desflurane group(n=45)	Delirium occurred in 1 (p=0.00695) patient in the propofol group and 0 (P =0.00695) patient in the desflurane group, there was no significant difference between them (P= 0.315 > 0.05)

### 6.2 General anesthesia and non-general anesthesia

Non-general anesthesia includes local anesthesia, spinal anesthesia, and nerve blocks. Spinal anesthesia, also known as half-body anesthesia, is further categorized into epidural anesthesia and subarachnoid anesthesia depending on the site of drug injection. Nerve blocks include common procedures such as brachial plexus block for upper limb surgery and cervical plexus block for neck surgery. Nowadays, ultrasound-guided nerve block techniques are widely used for adjunctive analgesia and reduction of anesthesia drug consumption. In this paper, we focused on comparing the incidence of POD after non-general anesthesia versus general anesthesia. Through an in-depth analysis of these data, we aim to provide conclusive evidence to support the selection of appropriate anesthesia methods in clinical practice. Our results will help guide medical decisions, ensure patient safety, and optimize surgical outcomes.

A randomized controlled trial analyzed the incidence of POD between subarachnoid anesthesia and general anesthesia in hip fracture surgery on patients aged 50 and above, the results showed no association between the type of anesthesia and the occurrence of POD (80). Another identical study made the same comparison and came to the same conclusion that among the 633 participants in the subarachnoid anesthesia group, 130 (20.5%) experienced delirium, while among the 629 patients in the general anesthesia group, 124 (19.7%) experienced delirium, with a relative risk of 1.04 (95% CI, 0.84 to 1.30), indicating no significant statistical difference in the occurrence of cognitive dysfunction between the two anesthesia methods (81). This finding is consistent with the conclusion of the study by Tzimas et al. (82). Additionally, a study comparing patients aged 65 and above who received hip fracture surgery found that compared to general anesthesia, subarachnoid anesthesia, epidural anesthesia, or a combination of the two did not significantly reduce the incidence of POD (83). Similarly, studies related to hip surgery have shown similar results (84–86). Furthermore, a study evaluated the effectiveness of T12 Erector Spinae Plane Block (ESPB) in elderly patients undergoing lumbar spine surgery. Although bilateral T12 ESPB reduced the Numerical Rating Scale (NRS) scores within 48 hours after lumbar spine surgery and decreased perioperative opioid consumption, it did not significantly reduce the incidence of POD. In the ESPB group, 7 cases (6.7%) had POD, while in the control group, 10 cases (9.3%) had POD, with no statistically significant difference (P > 0.05) (87).

In contrast to the above-mentioned block anesthesia methods, limited evidence suggests that only nerve blocks, among local anesthesia techniques, may reduce the incidence of delirium and are associated with shorter hospital stays, improved pain management, and decreased inflammation (88–90). In a study comparing ultrasound-guided multi-nerve blocks (myofascial block, lumbar plexus block, and femoral nerve block) with general anesthesia in elderly patients with hip fractures, satisfactory intraoperative pain management and reduced early postoperative cognitive impairment were achieved, making it a potential alternative anesthesia method for elderly hip fracture patients (91). Another meta-analysis of nine randomized controlled trials involving 1,225 patients found that perineural analgesia reduced postoperative pain scores and/or opioid consumption. In patients undergoing major surgery, perineural analgesia may prevent POD (92).

Although some studies have provided strong evidence to support the role of peripheral block anesthesia and peripheral analgesia in the prevention of POD, larger sample-size studies are needed to verify the universality and reliability of their effects. In addition, these studies should include longer-term follow-up to more accurately assess long-term cognitive function outcomes (**Table 4**).

**Table 4:** Comparison of the effect of general and non-general anesthesia on POD.

Study	Year	Design	No. in cohort	Study protocol	Comment
Kyra O’Brien et al. (80)	2023	RCT	578 participants	Spinal anesthesia group (n=295) VS general anesthesia (n=286)	new or worsened delirium occurred in 100/295 (33.9%) versus 107/283 (37.8%; odds ratio [OR] 0.85; 95% confidence interval [CI] 0.60 to 1.19)
Mark D. Neuman et al. (81)	2021	RCT	1445 participants	Spinal anesthesia group (n=633) VS general anesthesia (n=629)	Delirium occurred in 130 of 633 patients (20.5%) in the spinal anesthesia group and 124 of 629 (19.7%) in the general anesthesia group (relative risk, 1.04; 95% CI, 0.84 to 1.30).
Petros Tzimas et al. (82)	2018	RCT	70 participants	general anesthesia (GA group=33) VS subarachnoid (spinal) anesthesia (S group=37)	Postoperative delirium was present in four patients (12%) for the GA group and 10 patients (27%) for the group receiving subarachnoid anesthesia.
Li Ting et al. (83)	2022	RCT	950 participants	regional anesthesia (spinal, epidural, or both techniques combined with no sedation; n = 476) VS general anesthesia (intravenous, inhalational, or combined anesthetic agents; n = 474).	Postoperative delirium occurred in 29 (6.2%) in the regional anesthesia group vs 24 (5.1%) in the general anesthesia group (unadjusted risk difference [RD], 1.1%; 95% CI, -1.7% to 3.8%; P = .48; unadjusted relative risk [RR], 1.2 [95% CI, 0.7 to 2.0]; P = .57]).
Kateryna Bielka et al. (84)	2021	RCT	88 participants	Group 1: Ultrasound-guided lumbar septal obstruction and sciatic nerve block (n=30) VS Group 2: spinal anesthesia (n=29) Group 3: general sevoflurane inhalation anesthesia (n=29)	The incidence of postoperative delirium in the three groups was respectively: Group 1: 0/30 Group 2: 1/29 Group 3: 1/29 p>0,05
Sandeep Bhushan et al. (85)	2022	meta-analysis	Eight studies including 3555 elderly patients	regional anesthesia (RA) VS general anesthesia (GA)	there was no significant difference in the prevalence of POD or POCD between RA and GA at 24 h [OR 0.73; 95% coincidence interval (CI) 0.19, 2.71, I2 = 53%; n = 452; P = 0.63],
Ajia Zhang et al. (87)	2023	RCT	230 participants	T12 erector spinal plane block(n= 115) VS control groups (n = 115)	Seven patients (6.7%) developed POD in the ESPB group and ten patients (9.3%) in the control group, without any statistically significant difference (P > 0.05).
Jue Gu et al. (91)	2021	RCT	94 participants	Group N received ultrasound-guided multiple nerve blocks(n= 47) VS Group G received general anesthesia(n = 47)	Early moderate delirium (24 h postoperatively) was significantly higher in group G than in group N.
Lu Wang et al. (92)	2023	Meta-analysis	A total of 1,225 patients from 9 RCTs	–	The incidence of POD [Odds Ratio (OR) = 0.48, 95% CI 0.32, 0.72; p = 0.0004; I2 = 0%] was significantly reduced in the PVB (paravertebral block) group.

### 6.3 Anesthesia depth

New evidence in the field of anesthesia indicates a significant impact of anesthesia depth on the occurrence of POD, which is a crucial determinant of patient outcomes (93). Anesthesia depth is reflected by the processed electroencephalogram (PEEG)-guided bispectral index (BIS) monitoring (94). According to the literature reviewed, most studies support a lower incidence of POD in anesthesia guided by BIS monitoring than under routine care (95–99). However, conflicting results have been reported in a randomized clinical trial where 1,232 patients were randomly assigned in a 1:1 ratio to receive anesthesia guided by PEEG (n=614) or routine anesthesia care (n=618). The findings showed no significant difference in the incidence of delirium between groups, with 157 patients (26.0%) experiencing POD in the guidance group of 604 patients, and 140 patients (23.0%) experiencing delirium in the routine care group of 609 patients (difference 3.0% [95% CI, -2.0% to 8.0%]; P = 0.22) (100). Considering the presence of non-objective factors, it can still be considered that intraoperative PEEG-guided monitoring anesthesia can prevent POD occurrence. Even more surprising, better control of anesthesia depth has been found to significantly prevent the occurrence of delirium, as many randomized clinical trials have categorized patients into light and deep anesthesia states to evaluate the incidence of POD, consistently showing a significant reduction in POD with lighter anesthesia (101, 102). The new strategies proposed in this study provide innovative insights for preventing POD occurrence, which not only enriches our understanding of the field but also provides a valuable reference for future research and practice (**Table 5**).

**Table 5:** Comparison of the effect of anesthesia depth on POD.

Study	Year	Design	No. in cohort	Study protocol	Comment
Matthew T V Chan et al. (95)	2013	RCT	921 participants	462 patients received BIS-guided anesthesia, and 459 patients were randomly assigned to the usual care group.	BIS-guided anesthesia reduced the risk of POCD 3 months after surgery.
F M Radtke et al. (96)	2013	RCT	1277 participants	BIS guided anesthesia group (n=638), control group (n=639)	Postoperative delirium was detected in 95 patients (16.7%) in the intervention group compared with 124 patients (21.4%) in the control group (P=0.036).
Yumei Zhou et al. (97)	2018	A Prospective Controlled Study	81 participants	The BIS group (n=41) VS the non-BIS group (n=40)	Delirium was significantly lower in the BIS group compared with the non-BIS group (17.5% vs. 27.5%) (P<0.001)
Matthew Sumner et al. (98)	2023	meta-analysis	Nine studies, which included 4648 eligible subjects	–	The incidence of POD in the pEEG-guided general anesthesia or lighter pEEG target group was 19.0% (440/2310) compared with 23.3% (545/2338) in the usual care or deeper pEEG target group (pooled odds ratio=0.78; 95% confidence interval, 0.60-1.00; P=0.054).
Berta Pérez-Otal (99)	2022	RCT	200 participants	The visible BIS group (n=98) versus the hidden BIS group (n=102)	Patients who developed delirium (n = 69) were significantly lower in the visible BIS group (n = 27; 39.1%) than in the hidden BIS group (n = 42, 60.9%; p = 0.043).
Troy s Wildes et al. (100)	2018	RCT	1232 participants	EEG-guided anesthetic administration (n =614) VS usual anesthetic care (n = 618).	Delirium occurred in 157 of 604 patients (26.0%) in the guided group and 140 of 609 patients (23.0%) in the usual care group (difference, 3.0% [95% CI, -2.0% to 8.0%]; P =0.22).
Frederick E Sieber et al. (101)	2010	RCT	114 participants	the deep sedation (BIS≈ 50) (n=57) VS the light sedation (BIS≥80) (n=57)	The prevalence of postoperative delirium was significantly lower in the light sedation group (11/57 [19%] vs 23/57 [40%] in the deep sedation group; P=0.02),
Zhu Shuxing et al. (102)	2023	RCT	226 participants	Propofol lighter sedation (n=57); Propofol heavier sedation (n=56); Dexmedetomidine lighter sedation (n=57); Dexmedetomidine heavier sedation (n=56);	In the propofol group, heavier sedation had a higher rate of POD (32.7% [18/55] vs the lighter sedation group (14.5% [8/55]; Risk ratio, 2.25; 95% CI, 1.069 to 4.736; p=0.025).

## 7 Perioperative drugs

A thorough examination of preventive measures for POD requires a comprehensive exploration of pharmacological interventions. However, the efficacy of pharmacological interventions for preventive pharmacotherapy of delirium remains uncertain due to inconclusive evidence currently available (103). The pathophysiological basis of delirium described earlier emphasizes the presumed efficacy of pharmacological agents. Potential therapeutic interventions may include reducing exposure to drugs with prominent anticholinergic properties (104), addressing and mitigating direct brain injury caused by infectious etiologies or drug toxicity, and alleviating physiological stress responses through the use of corticosteroids, benzodiazepines, dexmedetomidine, or melatonin. Looking ahead, this paper will focus on an in-depth analysis of drugs that have been extensively studied in contemporary research, as well as a comparative evaluation of these drugs.

### 7.1 Dexmedetomidine

Dexmedetomidine is a highly selective α-2 adrenoceptor agonist with a range of pharmacological properties, including sedation, anxiolysis, and mild analgesia, which contribute to its therapeutic efficacy in clinical settings (105). It has a favorable impact on cerebral hemodynamics characterized by a reduction in cerebral blood flow while maintaining coupling between cerebral blood flow and metabolism (106). Additionally, dexmedetomidine has been found to have opioid-sparing effects and enhance the analgesic effects of concomitant opioid medications (107).

In a prospective randomized controlled study, elderly patients 65 years or older undergoing total hip replacement under lumbar anesthesia combined with T12 paravertebral block were randomly assigned to receive supplemental propofol or dexmedetomidine sedation, the conclusion drawn was that intraoperative dexmedetomidine sedation may be associated with a lower incidence of POD (108). In another randomized clinical trial, 226 patients aged 65 or above undergoing hip joint surgery were randomly divided into four groups: propofol light sedation group, propofol deep sedation group, dexmedetomidine light sedation group, and dexmedetomidine deep sedation group. The occurrence of delirium was the primary outcome, and the same conclusion was reached: compared to propofol, dexmedetomidine had a lower incidence of delirium in elderly patients with hip joint fractures (102). In evaluating the relationship between POD and commonly used sedatives such as sevoflurane, desflurane, isoflurane, dexmedetomidine, propofol, midazolam, and ketamine, Cui et al. found that dexmedetomidine was associated with a lower incidence of POD, while midazolam was associated with more POD cases (44). Numerous studies have demonstrated the association between dexmedetomidine and a low incidence of POD (109–113) Although dexmedetomidine holds promising potential as a treatment for POD, further research is needed to establish its undeniable efficacy. Additionally, vigilance regarding adverse reactions is necessary, considering reports of cardiovascular events such as hypertension, tachycardia, myocardial ischemia, cerebrovascular accidents, and hypoxemia (114). Prospective studies should focus on the specific needs of elderly patients with hip fractures to elucidate an optimized treatment strategy that maximizes therapeutic benefits while minimizing risks (**Table 6**).

**Table 6:** Comparison of the effect of dexmedetomidine on POD.

Study	Year	Design	No. in cohort	Study protocol	Comment
Bin Mei et al. (108)	2018	RCT	296 participants	Propofol groups (n=148) (P grpup) VS dexmedetomidine groups (n=148) (D grpup)	Compared to Group P, there was a smaller proportion of patients in Group D who exhibited POD (odds ratio [OR]: 0.41; 95% CI: 0.20-0.88; P = 0.03).
Mustafa S Aydogan et al. (109)	2013	RCT	42 participants	dexmedetomidine (DEX) groups (n=22) VS midazolam (MDZ) groups (n=20)。	Delirium was significantly higher in the group MDZ than in group DEX (31.3% vs 12.5%) when analyzed as the endpoint of CAM-ICU (P < 0.05).
X L Wang et al. (110)	2018	RCT	160 participants	ropivacaine and dexmedetomidine (n=80) VS only ropivacaine controlled group (n=80)	The incidence of 3-day delirium was lower in the dexmedetomidine group (5%) than in the controlled group (15%) (χ2 = 4.444, P<0.05).
Zhu Shuxing et al. (102)	2023	RCT	226 participants	Propofol lighter sedation (n=57); Propofol heavier sedation (n=56); Dexmedetomidine lighter sedation (n=57); Dexmedetomidine heavier sedation (n=56);	There was a significant association between dexmedetomidine and a lower incidence of delirium (11.9% [13/109] vs the propofol group (23.6% [26/110]; Risk ratio, 0.51; 95% CI, 0.274 to 0.929; p=0.024).
Bin Mei et al. (111)	2020	RCT	366 participants	Propofol groups (n=183) (P组) VS dexmedetomidine groups (n=183) (D组)	The incidence of POD in Group D was lower than in Group P (odds ratio[OR]: 0.54; 95% CI: 0.31-0.92, P=0.032).
Hyun-Jung Shin et al. (112)	2022	RCT	732 participants	Propofol (n = 366) groups VS Dexmedetomidine (n = 366) groups	postoperative delirium was significantly lower in the dexmedetomidine group than in the propofol group (3.0% v. 6.6%; odds ratio, 0.42;95% CI,0.201to 0.86; p= 0.036).
Niu Jing-Yi et al. (113)	2023	RCT	150 participants	intravenous dexmedetomidine groups (n=49), intranasal dexmedetomidine groups (n=50) and intratracheal dexmedetomidine groups (n=49)	Compared with the intranasal group, the intravenous group had a significantly lower occurrence of POD within 3 days (3 of 49 [6.1%] vs 14 of 50 [28.0%]; odds ratio [OR], 0.17; 95% confidence intervals [CIs], 0.05-0.63; P <.017). Meanwhile, patients in the intratracheal group had a lower incidence of POD than those in the intranasal group (5 of 49 [10.2%] vs 14 of 50 [28.0%]; OR, 0.29; 95% CI, 0.10-0.89; P <.017). Whereas, there was no difference between the intratracheal and intravenous groups (5 of 49 [10.2%] vs 3 of 49 [6.1%]; OR, 1.74; 95% CI, 0.40-7.73; P >.017).

### 7.2 Benzodiazepines

As anxiolytics, sedatives, and hypnotics, benzodiazepines have significant efficacy and possess anticonvulsant and central muscle relaxant effects (115). The use of these compounds in the preoperative stage contributes to alleviating preoperative anxiety, enhancing anesthesia effects, reducing the required dose of anesthetic agents, improving perioperative safety, and promoting postoperative amnesia for intraoperative adverse stimuli (116). Monitoring studies in the United States have indicated that 80% of patients undergoing orthopedic surgery receive benzodiazepines during the perioperative period, despite the associated risks of POD and delayed neurocognitive recovery (115). It is important to recognize that the use of benzodiazepines often synergistically increases the consumption of opioids, with their influence extending to perioperative analgesia and indirectly impacting the manifestation of POD.

A retrospective cohort study by the American Geriatrics Society investigated the incidence of POD in patients with hip fractures and concluded that the use of long-acting benzodiazepines (OR 1.82, CI 1.74-1.89) and short-acting and long-acting benzodiazepines (OR 1.56, CI 1.48-1.63) was associated with an increased likelihood of POD (117). Similarly, an article from a national tertiary referral center in Thailand studying the relationship between cognitive dysfunction and selective major surgeries in elderly adults arrived at the same conclusion, considering benzodiazepines to be an independent predictive factor for the development of POD in elderly patients undergoing major surgeries (118). Cui et al. and Breschan et al. also hold a consistent view on this (44, 119). In terms of the use of benzodiazepines, a retrospective cohort study categorized patients receiving oral benzodiazepines into four groups: continued use, discontinued use, initiated use, and no use (never used). The outcome indicated that the abrupt cessation of perioperative benzodiazepines may be a risk factor for POD, thus suggesting a preventative approach to the clinical use of these drugs (120). Despite extensive research indicating an association between benzodiazepines and a higher incidence of POD, there still exist some conflicting views. Yang et al. randomly divided patients undergoing orthopedic surgery into two groups, one receiving remimazolam and the other receiving propofol anesthesia, and the results showed that, compared to propofol, remimazolam anesthesia was not associated with an increased incidence of POD in elderly orthopedic surgery patients (121). Another study investigating the impact of multimodal anxiety relief strategies, including oral melatonin or midazolam, on POD following sevoflurane anesthesia, found that the incidence of delirium in the midazolam group was similar to that of the placebo group (122).

The current literature shows that there are significant differences in the results of studies on the effect of benzodiazepines on POD incidence. Given this, caution is recommended against the use of this class of drugs in clinical practice, and further in-depth studies are carried out to clarify the mechanism of action and potential risks (**Table 7**).

**Table 7:** Comparison of the effect of benzodiazepines on POD.

Study	Year	Design	No. in cohort	Study protocol	Comment
Jashvant Poeran et al. (117)	2020	Retrospective Cohort Study	505152 participants		Increased odds for postoperative delirium were seen for long-acting benzodiazepines (OR 1.82, CI 1.74 to 1.89), the combined use of short and long-acting benzodiazepines (OR 1.56, CI 1.48 to 1.63)
Saranya Lertkovit et al. (118)	2022	A Prospective Study	250 patients (141 males, 109 females)		Intraoperative benzodiazepine as independent variables associated with the development of POCD. (adjusted OR [aOR]: 2.24, 95% CI: 1.10–4.68; p = 0.026)
Chie Omichi et al. (120)	2021	A Retrospective Cohort Study	250 participants	29(11.6%), 49(19.6%), 8(3.2%), and 164(65.6%) patients were classified into “Continued,” “Discontinued,” “Initiated,” and “Never-used” groups	Among 250 patients, 78 (31%) developed post-operative delirium. The Discontinuation group had a higher rate of delirium (49%, 24/49) than the other groups (Continuation [14%, 4/29]; Initiation [38%, 3/8], Never used [29%, 47/164], p = 0.008).
Jin-Jin Yang et al. (121)	2023	A Prospective Randomized Controlled Clinical Trial	300 participants	remimazolam group (n=147) VS propofol group (n=153)	POD occurred in 23 (15.6%) of 147 ramazolam patients and 19 (12.4%) of 153 propofol patients (RR, 1.26; 95% CI, 0.72 to 2.21; RD, 3.2%; 95% CI, −4.7% to11.2%; P = 0.42]).
Lily Singla et al. (122)	2021	RCT	132 participants	Melatonin group(n = 45), Midazolam group(n=43) and Placebo group(n=44)	Compared with placebo, the absolute risk of emergence delirium decreased significantly when premedicated with melatonin [absolute risk ratio = 23.3 (95% CI 3.7 to 42.9), P = 0.03], whereas it did not differ when premedicated with midazolam [absolute risk ratio = -5.8 (95% CI -26.8 to 15.1), P = 0.59

### 7.3 Painkiller

Acute nociceptive stimulation is a key link between preoperative cognitive impairment and postoperative cognitive dysfunction, particularly delirium (123). The paradigm of perioperative pain management typically involves the combination of opioid analgesics and non-steroidal anti-inflammatory drugs (NSAIDs). It is important to recognize that while opioid therapy may potentially induce delirium (124), inadequate management of pain itself is a stronger independent risk factor for delirium (125), as demonstrated in clinical investigations of subjects undergoing interventions for hip fracture. The incidence of delirium increases when opioids are omitted or administered at subtherapeutic doses. Notably, patients receiving suboptimal pain management, compared to those receiving adequate analgesia, have a 9-fold increased risk of delirium development, even when cognitive function is preserved. Thus, inadequate analgesia and inappropriate pain management patterns have been described as risk factors for delirium in frail elderly populations (126).

Relying solely on opioid analgesics may not be sufficient to effectively alleviate pain, especially following orthopedic surgery. Therefore, NSAIDs are commonly used as adjunctive analgesics to opioids postoperatively (127). Ibuprofen and other related NSAIDs are widely used to alleviate pain and inflammation in various clinical conditions. In contrast, opioid-mediated analgesia does not alleviate the inflammatory component of pain perception and carries the potential for serious adverse effects (128).

In a randomized, double-blind, multicenter trial, patients aged 60 or above undergoing elective total hip or knee arthroplasty were assigned to receive parecoxib or placebo in a 1:1 ratio, The results demonstrated that in a perioperative low-risk elderly population, the use of parecoxib as part of a multimodal analgesic regimen reduced the incidence of POD without increasing adverse outcomes when compared to intravenous morphine (129). Li et al. also concluded that parecoxib sodium analgesia reduced the incidence of POD and had a neuroprotective effect in elderly patients in a study of parecoxib sodium versus placebo-controlled hip replacement surgery (130). Regular intravenous administration of acetaminophen in combination with intravenous administration of propofol or dexmedetomidine is currently a high-quality strategy for reducing the frequency of POD (127). The study consistently concluded that multimodal analgesia strategies were effective in reducing the incidence of POD. This finding provides a clear guideline for the prevention of POD in clinical practice and has important practical significance (**Table 8**).

**Table 8:** Comparison of the effect of Painkiller on POD.

Study	Year	Design	No. in cohort	Study protocol	Comment
Dong-Liang Mu et al. (129)	2017	RCT	620 participants	placebo group(n=310) VS parecoxib group(n=310)	The incidence of delirium was significantly reduced from 11.0% (34/310) with placebo to 6.2% (19/310) with parecoxib (relative risk 0.56, 95% confidence interval 0.33-0.96, P = .031).
Jing-zhu Li et al. (130)	2013	RCT	80 participants	control group (group C, n = 40) and parecoxib group (group P, n = 40)	Compared with group C, the additional amount of morphine, postoperative time, rate of POD at T1-T4, and the level of NSE at t2-t5 and S-100β at t1-t5 were lower in group P (P < 0.05).

### 7.4 Steroid

The immune-inflammatory pathway is associated with the pathogenesis of POD in patients with hip fractures (131), and corticosteroids have potent anti-inflammatory properties. The use of anti-inflammatory therapy in the prevention and treatment of delirium has been a strong research topic (132). Studies have shown that preoperative single-dose low-dose dexamethasone can reduce the incidence of POD in elderly orthopedic surgery patients (133, 134), with the greatest reduction observed in the absence of general anesthesia (135). Furthermore, compared to patients receiving low-dose glucocorticoids, those receiving high-dose glucocorticoids have a lower incidence of POD (136).

However, contradictory views to the above are still present. In patients with hip fractures, there was no significant difference in the severity of delirium scores between the dexamethasone and placebo groups, There was no difference in the incidence of delirium between the groups: 6/40 (15%) in the dexamethasone group and 9/39 (23%) in the placebo group (137). In addition, a meta-analysis indicated that prophylactic dexamethasone did not reduce the incidence of wound infection and POD (138). To advance research in this area, more experiments are urgently needed to explore alternative strategies for preventing postoperative cognitive impairment.

At the same time, it is crucial to delve deeper into the pathophysiological mechanisms behind these symptoms, which will help us understand POD more fully and develop more effective preventive measures (**Table 9**).

**Table 9:** Comparison of the effect of steroid on POD.

Study	Year	Design	No. in cohort	Study protocol	Comment
Jian-Wen Huang et al. (133)	2023	RCT	160 participants	the dexamethasone group (n=80) and the placebo group (n=80)	The dexamethasone group had a lower incidence of POD than the placebo group within the first 5 days after surgery [ (9/80, 11.3% vs. 21/80, 26.3%, RR = 0.83, 95% CI 0.71-0.97, P = 0.015].
C G Clemmesen et al. (134)	2018	RCT	117 participants	methylprednisolone group (n=59) or placebo. group (n=58)	The prevalence of postoperative delirium (10/59 vs. 19/58, p = 0.048)was significantly lower in the methylprednisolone compared with the placebo group.
Hussein Nasser Awada et al. (136)	2022	RCT	53 participants	High-dose (HD)dexamethasone group (n=26) VS low-dose (LD)dexamethasone group (n=27)	A lower incidence of POD occurred in the HD group (n = 0, 0%) versus LD (n = 5, 19%)
M T Kluger et al. (137)	2021	RCT	79 participants	dexamethasone group (n=40) VS placebo. Group (n=39)	Delirium incidence did not differ between groups: 6/40 (15%) in the dexamethasone group vs. 9/39 (23%) in the placebo group, relative risk (95%CI) 0.65 (0.22-1.65), p = 0.360).
Li Li-Qin et al. (138)	2019	Meta-analysis	Five RCTs		There was no significant difference between the dexamethasone group and the placebo group in terms of the incidence of POCD 30 days after surgery (RR [relative risk] 1.00; 95% CI [confidence interval: 0.51, 1.96], P = 1.00, I2 = 77%) or the incidence of POD (RR 0.96; 95% CI [0.68, 1.35], P = 0.80, I2 = 0%).

### 7.5 Antipsychotic drug

Antipsychotic treatment can generally be divided into two classes: classic first-generation antipsychotics (FGAs), also known as typical antipsychotics, mainly including chlorpromazine and haloperidol, and more modern second-generation antipsychotics (SGAs), commonly known as atypical antipsychotics, including quetiapine, risperidone, and olanzapine. Many researchers have studied the use of antipsychotic medications for the prevention of POD, and the results have been highly encouraging.

Haloperidol is a typical antipsychotic that primarily exerts its antipsychotic properties by selectively antagonizing dopamine/antipsychotic receptors in the brain, leading to alterations in dopamine turnover (139). Its clinical application extends to the treatment of schizophrenia and bipolar affective disorder (140), emphasizing its effective blockade of extrapyramidal dopamine and significant antiemetic effects (141). Fukata’s article is a good demonstration of this point of view (142). Nevertheless, in two randomized controlled clinical studies that compared haloperidol with placebo, prophylactic haloperidol use did not have a significantly better effect on POD incidence than placebo (143, 144). However, in elderly orthopedic patients undergoing knee or hip arthroplasty, preoperative oral administration of low-dose quetiapine one hour before surgery can reduce the occurrence of POD, improve postoperative sleep quality, and increase satisfaction with postoperative pain management at 24 hours (145).

In cardiac surgery, two studies have shown that the use of intraoperative risperidone is associated with a lower incidence of POD compared to placebo (11.1% vs. 31.7%, P=0.009, relative risk = 0.35, 95% confidence interval [CI] = 0.16-0.77) (146, 147). As an atypical antipsychotic, olanzapine significantly reduces the incidence of delirium when orally administered at 10 mg during the perioperative period. These findings suggest that olanzapine may be an effective strategy for preventing POD (148). However, despite the use of olanzapine for prophylaxis, a proportion of patients may still experience delirium. Risk reduction for delirium can be achieved through prophylactic administration of olanzapine, optimization of perfusion and oxygenation, and limiting intraoperative opioid use (149).

Taken together, we believe that these findings provide a new perspective for the future clinical use of antipsychotics and are expected to guide clinicians more effectively to prevent POD. To ensure the reliability of these conclusions, more research is recommended to verify the accuracy and clinical applicability of these findings (**Table 10**).

**Table 10:** Comparison of the effect of antipsychotic drugs on POD.

Study	Year	Design	No. in cohort	Study protocol	Comment
Shinji Fukata et al. (142)	2017	RCT	201	The haloperidol group (n = 101) VS the control group (n = 100)	The incidence of severe postoperative delirium in the intervention group (18.2%) was significantly lower than that in the control group (32.0%) (p = 0.02).
Kees J Kalisvaart et al. (143)	2005	RCT	430	The haloperidol group (n = 215) VS the control group (n = 215)	The percentage of patients with postoperative delirium in the haloperidol and placebo treatment condition was 15.1% and 16.5%, respectively (relative risk=0.91, 95% confidence interval (CI)=0.6-1.3);
Babar A Khan et al. (144)	2018	RCT	135	The haloperidol group (n = 68) VS the control group (n = 67)	No significant differences were observed between those receiving haloperidol and those receiving placebo in incident delirium (n=15 (22.1%) vs n=19 (28.4%); p = .43),
Yang Yongping et al. (145)	2023	RCT	111	control group (n=54) VS quetiapine group (n=57)	The incidence of POD at 24 h after surgery in group Q was lower than that in group C (P=0.036)
U Prakanrattana et al. (146)	2007	RCT	126	risperidone group (n=63) VS control group (n=63)	The incidence of postoperative delirium in the risperidone group was lower than in the placebo group (11.1% vs. 31.7% respectively, P=0.009, relative risk = 0.35, 95% confidence interval [CI] = 0.16-0.77).
Sameh M Hakim et al. (147)	2012	RCT	101	risperidone group (n=51) VS control group (n=50)	Seven (13.7%) patients in the risperidone group experienced delirium versus 17 (34%) in the placebo group (P = 0.031)
Kenneth A Larsen et al. (148)	2010	RCT	400	olanzapine group (n=200) VS control group (n=200)	The incidence of delirium was significantly lower in the olanzapine group than in the placebo group;

### 7.6 Melatonin

Melatonin has a regulatory effect on the circadian rhythm and, due to its role in synchronizing circadian rhythms, it can improve sleep onset and enhance sleep quality. This neurohormone exhibits flexibility in modulating excitatory processes in the central nervous system. Reduced melatonin secretion has been associated with increased susceptibility to delirium, particularly in the elderly and individuals with cognitive impairments. In terms of the pathophysiology of delirium, melatonin has shown efficacy in alleviating oxidative stress in various conditions (150). Its mechanisms include direct neutralization of reactive oxygen and nitrogen species, as well as indirect promotion of antioxidant enzymes and inhibition of pro-oxidant catalysts (151). Furthermore, melatonin is involved in sequestering transitional metals (152) and reducing the formation of harmful hydroxyl radicals, thereby mitigating oxidative stress (153). Its anti-free radical properties further extend to reducing the side effects of various medications and methamphetamine, providing the biological basis for the use of melatonin in preventing delirium (154, 155).

In a meta-analysis analyzing the incidence of POD in elderly surgical patients using melatonin or ramelteon as preventive measures compared to placebo, the occurrence of delirium in the placebo group ranged from 4% to 33% and was reduced to 0% to 30% in the melatonin group, with an odds ratio of 0.63 (95% CI, 0.46-0.87; P = 0.006) (156). Kain et al. believed that the incidence of delirium in the melatonin group was lower (P < 0.05), and the effect was dose-dependent.; the incidence rates after administration of melatonin at 0.05 mg/kg, 0.2 mg/kg, and 0.4 mg/kg were 25.0%, 8.3%, and 5.4%, respectively (157). A multimodal anti-anxiety approach, including oral melatonin, significantly reduced the emergence of delirium following sevoflurane anesthesia (122). While most studies suggest that melatonin may effectively reduce the incidence of POD (158, 159), in studies of cardiac surgery, the prophylactic use of melatonin did not show a statistically significant impact on POD (160). Furthermore, there is limited evidence that ramelteon is not effective in preventing POD (161, 162). Although the current findings diverge somewhat, they collectively highlight the importance of cross-disciplinary clinicians considering drug combination therapy in practice. This combined treatment strategy may help improve the effectiveness of prevention of POD and therefore deserves further exploration and validation in future clinical practice (**Table 11**).

**Table 11:** Comparison of the effect of melatonin on POD.

Study	Year	Design	No. in cohort	Study protocol	Comment
Zeev N Kain et al. (157)	2009	RCT	140	oral midazolam 0.5 mg/kg (n=35); oral melatonin 0.05 mg/kg (n=35); oral midazolam 0.2 mg/kg (n=35); oral midazolam 0.4 mg/kg (n=35)	Children who received melatonin developed less emergence delirium compared with those who received midazolam (P < 0.05), and the effect was dose-related; the incidence after 0.05 mg/kg melatonin was 25.0%, incidence after 0.2 mg/kg melatonin was 8.3%, and incidence after 0.4 mg/kg melatonin was 5.4%.
Daisuke Hokuto et al. (158)	2020	RCT	306	Ramelteon group (n=120) VS control group (n=186)	The incidence of postoperative delirium was significantly lower in the ramelteon group (5.8% vs. 15.1%, P = 0.035).
shi Yicheng et al. (159)	2021	RCT	297	Placebo group ( (n = 149) VS melatonin group (Mel) (n= 148)	The incidence of postoperative delirium was significantly lower in the Mel group than in the placebo group (27.0% vs. 39.6%, respectively, P = 0.02).
Ashley M Campbell et al. (156)	2019	Meta-Analysis	6 RCTs		The incidence of delirium ranged from 0 to 30% in the intervention groups versus 4-33% in the comparator groups and was significantly reduced in the melatonin group, with a summary effect of the meta-analysis yielding an odds ratio of 0.63 (95% CI 0.46 to 0.87; 0.006; I2 = 72.1%).
Andrew H Ford et al. (160)	2020	RCT	210	melatonin group (n=105) VS placebo (n=105)	(melatonin 21/98, 21.4%; placebo 21/104, 20.2%; adjusted odds ratio [OR] = .78; 95% confidence interval [CI] = .35-1.75).
Mariko Kinouchi et al. (161)	2023	RCT	108	Ramelteon group (n = 55) VS placebo group (n = 53)	There is no significant difference in preventing postoperative delirium between ramelteon and placebo (χ2 = 0.30, degrees of freedom = 1, p = 0.60)
Esther S Oh et al. (162)	2020	RCT		Ramelteon group (8 mg) (n=33) VS placebo group (n=28)	Delirium incidence during the 2 days following surgery was 7% (5 of 71) with no difference between the ramelteon versus placebo: 9% (3 of 33) and 5% (2 of 38), respectively.

### 7.7 Cholinergic agonists

A large body of literature strongly indicates that alterations in cholinergic neurotransmission are associated with the occurrence of POD (163, 164). Genetic polymorphisms in cholinergic receptors, particularly the CHRM2 and CHRM4 genes (165), may support this relationship. While theoretically, interventions aimed at increasing acetylcholine (ACh) levels through the use of acetylcholinesterase inhibitors are considered potential interventions, existing controlled studies evaluating the efficacy of this treatment approach have yielded inconsistent results. Young et al. and Massoudi et al. supported this view, suggesting that sustained action of acetylcholine could significantly reduce the incidence of POD (166, 167). Other articles argue otherwise. A study found that administration of scopolamine within 24 hours after induction of anesthesia did not significantly change the incidence of POD (168). In two randomized controlled trials of acetylcholinesterase inhibitors, the authors randomly assigned patients to both the acetylcholinesterase inhibitor group and the control group, and they found surprisingly consistent results that there was no statistical difference in the incidence of POD between the control group and the acetylcholinesterase inhibitor (169, 170). This finding contradicts the idea that there is an association between cholinergic neurotransmission alterations and POD occurrence. To ensure the accuracy and reliability of the study, more studies are recommended to delve into the mechanism of action of the cholinergic system in POD occurrence and verify this paradoxical phenomenon.

Based on current research, analgesics are effective in preventing the development of POD. However, there is a discrepancy in the literature regarding the efficacy of alternative anesthetic techniques and perioperative medications in POD prevention. These conflicting findings present new avenues and challenges for future research in POD prevention and clinical pharmacology. Future studies should aim to clarify these differences and provide more definitive guidance for clinical practice (**Table 12**).

**Table 12:** Comparison of the effect of Cholinergic agonists on POD.

Study	Year	Design	No. in cohort	Study protocol	Comment
Young Chul Youn et al. (166)	2017	RCT	62	rivastigmine group (n=31) VS placebo group (n=31)	Postoperative delirium occurred in 5 patients in the rivastigmine group and 13 patients in the control group (p = 0.013).
Nilofar Massoudi et al. (167)	2023	RCT	100	rivastigmine group (n=50) VS placebo group (n=50)	Treatment with Rivastigmine was significantly associated with reduced day one post-op delirium, (Odds Ratio (OR) = 0.35, 95% Confidence Interval (CI) 0.11 to 0.97, p = 0.05)
Claudia D Spies et al. (168)	2021	RCT	261	Physostigmine group (n=130) VS placebo (n = 131)	The incidence of POD did not differ significantly between the physostigmine and placebo groups (20 versus 15%; P = 0.334).
Fanghao Liu et al. (169)	2022	RCT	401	Neostigmine group (group N) (n=196) and placebo group (group P) (n=205)	The incidence of POD was 20.20% (81/401), including 19.39% (38/196) in group N and 20.98% (43/205) in group P. There was no significant statistical significance in the incidence of POD between group N and group P (P > 0.05)
Benjamin Liptzin et al. (170)	2005	RCT	80	Donepezil group (n=40) VS placebo group (n=40)	Delirium was found on at least 1 postoperative day in 18.8% of subjects, but there were no significant differences between the donepezil and placebo groups

## 8 Prevention for POD

In 2015, the American Geriatrics Society disseminated an investigative synopsis addressing the incidence of POD amongst the geriatric demographic. The report delineated three pivotal non-pharmacologic directives: (i) standardization of educational initiatives within health-care institutions; (ii) implementation of multifaceted, non-pharmacological prophylaxes for individuals identified as high-risk, under the vigilant oversight of an interdisciplinary cohort; (iii) comprehensive interventions for patients with a confirmed diagnosis. Furthermore, the report underscored the dearth of robust evidence endorsing the utilization of technological apparatuses in management (171).

Preventive strategies, coupled with prompt recognition, are deemed fundamental in forestalling POD after hip fracture surgeries. Medical practitioners during the perioperative timeline should maintain heightened vigilance for POD onset, especially in patients presenting with both intrinsic and extrinsic risk factors. Contemporary strides have yielded POD risk assessment models (172, 173), instrumental in the stratification of delirium risk among this patient cohort, thereby guiding the formulation of preventative strategies.

**Figure 3:**
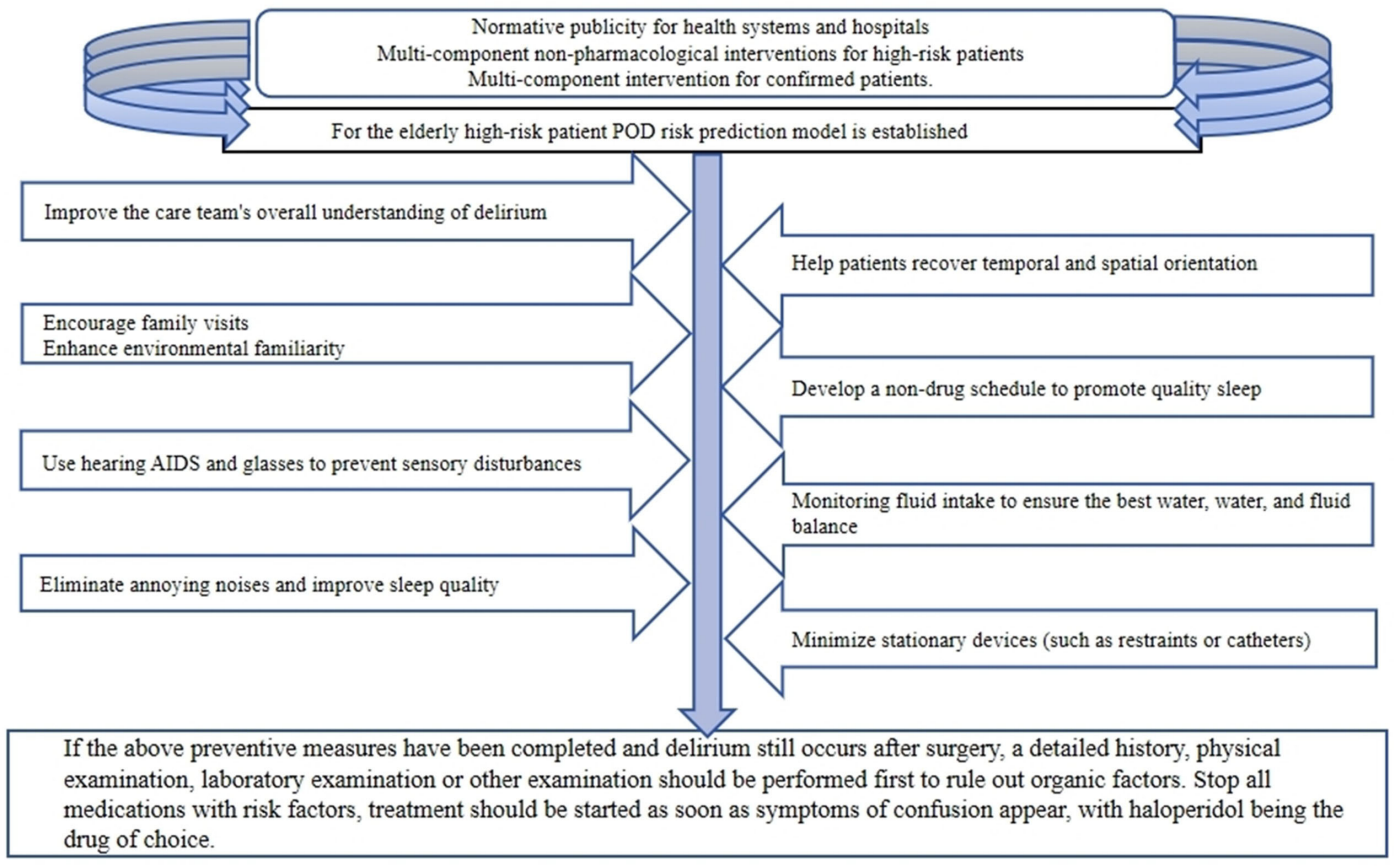
Strategies for the non-pharmacological mitigation of delirium and subsequent therapeutic interventions upon detection: a holistic approach to patient care.

Healthcare proxies often serve as initial observers of behavioral shifts in post-surgical patients; hence they must be adequately trained to recognize the indicators of POD at the earliest instance. Standard nursing interventions encompass the strategic placement of timekeeping devices and calendars to assist with temporal and spatial reorientation, fostering visitations by kin, establishing non-pharmacological routines to enhance sleep quality, delineating nocturnal rest from daytime activities, abating disruptive auditory stimuli, endorsing the utilization of sensory aids, assuring optimal hydration and nutritional status to mitigate malnutrition, and the vigorous management of pain (**Figure 3**). Furthermore, our analysis incorporated a comparison of geriatric orthopedic care methodologies. The geriatric orthopedic care paradigm integrates a multidisciplinary protocol, which encapsulates comprehensive assessments and optimization of the geriatric population post-orthopedic interventions. An abundance of research has verified that perioperative multimodal care substantially diminishes the incidence of POD (P <.00001) (174).

## 9 Conclusion

While the utilization of surgical anesthesia and perioperative pharmacotherapy presents an elevated predisposition towards the advent of POD in hip surgery, judicious regulation of dosage and meticulous selection of pharmacological agents can mitigate such risk. It is incumbent upon anesthesiologists and surgical care teams to perform a comprehensive assessment of the patient’s chronological age, surgical category, and pathophysiological status among other criteria, to formulate an optimal anesthetic plan that encompasses the judicious choice of drugs and techniques. Additionally, family members and healthcare providers must possess cognizance of the potential for postoperative cognitive dysfunction, actively engaging in preventive strategies to attenuate the incidence.

Postoperative cognitive dysfunction, notably delirium, represents a prevalent post-surgical complication in geriatric cohorts after femoral fractures. The application of anesthesia and ancillary perioperative medications requires heightened scrutiny. Healthcare practitioners must exercise a strategic approach in the preoperative and intraoperative phases, inclusive of a thorough appraisal of the patient’s health profile, leading to the strategic administration of anesthetic agents and perioperative medications, with the express intent of minimizing the manifestation of POD.
